# Is β-cell failure in type 2 diabetes mellitus reversible?

**DOI:** 10.4103/0973-3930.41978

**Published:** 2008

**Authors:** Rashmi Jain, Udaya Kabadi, M. Kabadi

**Affiliations:** Department of Endocrinology, University of Iowa College of Medicine, VAMC, Des Moines, IA, USA; 1 VAMC, Phoenix, AZ, USA

**Keywords:** Obese, type 2 diabetes, weight loss, β-cell function

## Abstract

**BACKGROUND:**

In the UK Prospective Diabetes Study (UKPDS), many subjects maintained glycemic goal (HbA_1c_ < 7.0%) at 9 years, showing that β-cell function was preserved and that the initial decline in β-cell function recovered with sulphonylureas. Moreover, obese subjects using high daily doses of insulin for several years rarely require insulin or oral hypoglycemic agents to maintain their glycemic goal following weight loss achieved by gastric bypass surgery. Thus, declining β-cell function during the course of type 2 diabetes mellitus (T2DM) is neither universal nor permanent.

**OBJECTIVE:**

To assess β-cell function in morbidly obese subjects before insulin withdrawal and on attaining the glycemic goal with weight loss and oral agents.

**MATERIALS AND METHODS:**

Serum C-peptide (CPEP) and glucose (G) concentrations were determined up to 180 min during an oral glucose tolerance test (OGTT) with 75 glucose in 10 obese men with T2DM, before insulin withdrawal, and on achieving the glycemic goal with metformin, glimepiride, and weight loss. Ten age-matched healthy men participated as controls. Cumulative responses (CR) of CPEP and G were calculated by adding differences between the level at each time-period during OGTT and fasting (F) concentration. β-Cell function was expressed as the FCPEP as well as the insulinogenic index (CRCPEP/CRG). Insulin sensitivity was determined as FCEP × FG.

**RESULTS:**

FCPEP was decreased, though still present, prior to insulin withdrawal. Moreover, on attaining the glycemic goal over 6-9 months, FCPEP, CRPEP/CRG, and FCPEP × FG improved markedly (*P* < 0.001).

**CONCLUSION:**

Decline in β-cell function in morbidly obese T2DM may not be progressive and is reversible on improving insulin sensitivity and on eliminating the inhibition by exogenous insulin.

## Introduction

β-Cell dysfunction and insulin resistance are known to be the two major mechanisms involved in the pathophysiology of type 2 diabetes mellitus (T2DM). The β-cell dysfunction is initially characterized by impairment in the first phase of insulin secretion following glucose stimulation, resulting in impaired glucose tolerance (a prediabetic state) and postprandial hyperglycemia.[1–3] As the disease progresses, the second phase secretion declines, resulting in fasting hyperglycemia, i.e., either impaired fasting glucose (IFG) or T2DM.[4] This β-cell dysfunction is thought to be progressive and irreversible. However in the UKPDS, many subjects achieved the glycemic goal (HbA_1c_ < 7.0%) at 9 years while being treated with oral monotherapy, denoting the lack of a progressive β-cell failure.[5,6] Moreover, β-cell function improved to 80% from the 50% seen at diagnosis following therapy with sulphonylureas.[7,8] Several other studies have also documented an improvement in insulin secretion after administration of sulfonylureas as well as diazoxide;[9–11] β-cell recovery is also noted with the weight loss that follows gastric bypass surgery in morbidly obese subjects with T2DM who have been using high-dose insulin for several years.[12–15] Finally, a recent study clearly demonstrated that β-cell failure in T2DM may be neither universal nor inevitable.[16] Therefore this study was conducted to determine the insulin secretion prior to and after withdrawal of exogenous insulin, while attaining desirable glycemic control (HbA_1c_ ≤ 7.0%) with initiation of oral hypoglycemic drugs as well as weight loss.

## Materials and Methods

Ten obese men with T2DM in the age range of 50-65 years and 10 healthy age-matched men participated in the study after signing the informed consent. The duration of DM was 10-15 years and all subjects were receiving insulin at doses of over 1.0 U/kg body weight, once or twice daily, for 2-10 years. Selection criteria included presence of morbid obesity, with BMI > 35 kg/m^2^; duration of diabetes of over 10 years and treatment with insulin alone for over 2 years, with the daily dose being more than 1.0 U/kg body weight; and a HbA_1c_ of over 8.0% on two successive determinations at an interval of 3-4 months prior to enrollment. Subjects with serum creatinine ≥1.5 mg/dl, AST and ALT ≥ 2.5 times the upper limit of normal laboratory values, hospitalization for any cause during the previous 6 months, unstable coronary artery disease, cerebrovascular disease, or peripheral vascular disease were excluded. Subjects with known mental instability, as well as those who refused to provide informed consent, were also excluded.

The demographics are shown in Table 1. The subjects were hospitalized for 30 days in the metabolic ward of the medical center. They received a 1200-1500-kcal American Diabetes Association (ADA) diet and were also subjected to daily exercise for 30 min, twice a day, in the physical therapy department. They were started on glimepiride 8 mg and metformin 850 mg daily; exogenous insulin was withdrawn simultaneously. Metformin was titrated at weekly intervals to a maximum of 2550 mg/day. In addition, dietary counseling was provided at weekly intervals by a registered dietician. The subjects were then followed at intervals of 4 weeks as outpatients, with recurrent counseling being given for compliance with diet, exercise, and oral agents. Serum C-peptide and glucose levels were determined after an overnight fast and up to 180 min during OGTT with 75 g glucose in subjects with T2DM prior to treatment (pre-treatment) and after attainment of HbA_1C_ < 7.0% with weight loss, metformin, and glimepiride (post-treatment); these values were also determined in the 10 healthy volunteers. Insulin and glucose responses during OGTT were assessed as cumulative responses as calculated by adding the differences between levels at each time period and fasting level. Cumulative response has been well documented to be a reliable expression of the integrated response as determined by the area under the curve over the duration of the OGTT.[17] Insulin secretion was expressed by fasting level of C-peptide (FCPEP) as well as an insulinogenic index as calculated by CRCPEP/CRG determined during OGTT.[18] Insulin sensitivity was expressed as a product of FG and FCPEP as recently documented.[19] Liver enzymes (i.e., ALT and AST), serum creatinine levels, and lipid profiles were also determined after an overnight fast, at the time of OGTT.

**Table 1:** Demographic characteristics of 10 men with type 2 DM and 10 normal subjects

	DM	Normal
Age (years)	59 ± 4	58 ± 5
Duration of DM (years)	13 ± 5	-
BMI (kg/m^2^)	43 ± 7	26 ± 2
Daily insulin dose (U/kg)	1.13 ± 0.8	-

## Results

**Figure 1:**
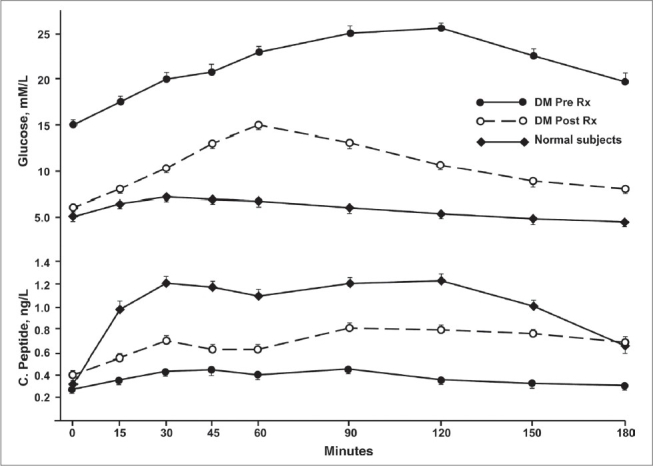
C-peptide response

At enrollment, serum creatinine and liver enzymes in subjects with T2DM were not significantly different from that in healthy volunteers and remained unchanged at the end of the study [Table 2]. Subjects with T2DM achieved a desirable glycemic control over a period of 6-9 months, as reflected by HbA_1c_ ≤ 7.0% [Table 3]. However, even with treatment the glycemia remained significantly higher in these subjects than in the normal healthy subjects [Table 3]. Insulin secretion improved markedly on achieving the desirable glycemic goal, as evidenced by the significant rises in FCPEP level as well as the insulinogenic index (CRCPEP/CRG) [Table 3]. Moreover, both early (within 30 min) and late (90-120 min) insulin secretion following glucose ingestion increased significantly, as shown by the C-peptide responses [Figure 1]. Finally, insulin sensitivity in the T2DM group also improved markedly following treatment, as assessed by the product of FG and FCPEP [Table 2]. However, neither the insulin secretory patterns nor the insulin sensitivity normalized despite the subjects attaining the desirable glycemic goal [Figure 1; Table 3]. Lipid profiles improved markedly in these subjects on achieving the desirable glycemic goal [Table 2].

**Table 2:** Comparison of pre-treatment and post-treatment hepatorenal function and lipids

	Pre-treatment	Post-treatment	Normal
Serum creatinine (mg/dl)	1.1 ± 0.2	1.0 ± 0.2	0.9 ± 0.2
AST (µ/ml)	30 ± 5	27 ± 5	26 ± 4
ALT (µ/ml)	22 ± 4	30 ± 6	30 ± 4
Total cholesterol (ng/dl)	210 ± 48^a^	170 ± 22^a^^b^	160 ± 19
Triglycerides (mg/dl)	277 ± 41^a^	142 ± 27^a^^b^	137 ± 13
HDLC (mg/dl)	32 ± 10^a^	47 ± 11^a^^c^	56 ± 9
LDLC (mg/dl)	116 ± 15^a^	97 ± 8^a^^c^	80 ± 7

a *P* < 0.05 *vs* normal

b *P* < 0.01 *vs* pre-treatment

c *P* < 0.05 *vs* pre-treatment

**Table 3:** BMI, HbA_1c_, fasting glucose (FG), fasting C-peptide (FCPEP), insulin sensitivity index (FG × FCPEP), and insulinogenic index during OGTT (CRCPEP/CRG) in 10 healthy men and in 10 morbidly obese subjects with type 2 DM prior to treatment (pre-treatment) and following attainment (post-treatment) of desirable glycemic goal (HbA_1C_ ≤ 7.0%) after weight loss and therapy with oral agents

	BMI (kg/m^2^)	HbA_1C_ (%)	FG (mM/l)	FCPEP (ng/l)	FG × FCPEP (mM.ng/l)	CRCPEP/CRG (ng/mM/l)
Pre-treatment	43 ± 7	9.6 ± 1.2	12.5 ± 0.9	0.28 ± 0.03	3.82 ± 1.0	0.02 ± 0.001
Post-treatment	33 ± 4^b^^d^	6.8 ± 0.1^a^^d^	6.2 ± 0.7^b^^d^	0.4 ± 0.06^b^^c^	2.28 ± 0.1	0.38 ± 0.11^b^^d^
Normal	24 ± 2	4.9 ± 0.1	5.3 ± 0.2	02.5 ± 0.03	1.26 ± 0.2	0.72 ± 1.21

a Cumulative response as determined by summation of differences between the level at each time period and basal level up to 180 min

b *P* < 0.01 *vs* pre-treatment

c *P* < 0.05 *vs* pre-treatment

d *P* < 0.05 *vs* normal

^e^*P* < 0.01 *vs* normal

## Discussion

This study demonstrates that reinitiation of oral agents and weight loss can help subjects in attaining the desirable glycemic goal without the need for insulin administration. This finding is consistent with previous observations that reinitiation of oral agents, including sulfonylureas, either reduced the daily insulin requirement or totally abolished the need for exogenous insulin.[20–26] Earlier studies have indicated that improvement in insulin sensitivity is the probable mechanism. However, the pattern of insulin secretion prior to and following reinitiation of oral agents and weight loss has not been well studied. This study clearly demonstrates the improvement in insulin sensitivity as well as enhancement of insulin secretion on achieving desirable glycemic control following withdrawal of insulin and reinitiation of oral agents and weight loss. The subnormal patterns of both insulin sensitivity and secretion despite achieving the desirable glycemic goal may be attributed to a lack of complete normalization of glycemia as well as persistence of obesity [Table 3]; in some studies, normalization of both the insulin secretory pattern and insulin sensitivity was documented in subjects with T2DM on achieving normal HbA_1c_ levels with weight loss and oral hypoglycemic drugs.[27–31] The markedly decreased β-cell function present at initial evaluation in our subjects may be attributed to suppression by long-term administration of exogenous insulin, especially in extremely high daily dose, as demonstrated in our recent study with various sufonylurea drugs.[32] Alternatively, the decline in β-cell function, especially in terms of postprandial insulin secretion, may also be attributed to extreme insulin resistance at the level of the β-cell itself in the presence of morbid obesity as has been described recently.[33,34]

The role of morbid obesity in the decline of both insulin secretion and sensitivity is further evident by the improvement in these parameters in subjects with T2DM following weight loss, exercise, and use of oral agents.[12–15,29–31] Even a moderate weight loss following a hypo-caloric diet is shown to improve insulin action and secretion.[28,30] Finally, the maintenance of a weight loss of 33% of body weight for more than 10 years, achieved by gastric bypass surgery, not only normalized glucose levels in patients with impaired glucose tolerance (IGT) or T2DM, but also improved hyperinsulinemia and the decreased insulin sensitivity.[12–15,29–31,35–38] Another similar study showed normalization of insulin sensitivity and restoration of a normal β-cell acute insulin response (AIR) to glucose ingestion, as well as a normal relationship of AIR to insulin sensitivity.[29] Therefore, the progressive β-cell failure documented in the UKPDS may also be attributed to increase in insulin resistance induced by the significant weight gain noted in all therapeutic arms of the study.[5,6,39] Moreover, in this study many subjects were at the glycemic goal (HbA_1C_ < 7.0%) at 9 years, denoting lack of β-cell failure.[5,6] Thus, the progressive β-cell failure was found to be neither universal nor inevitable in this study, a finding that was confirmed in another recent study.[16] Finally, we believe that the decline in β-cell function may be reversible, as documented in the UKPDS as well as in other studies following initiation of treatment with sulphonylureas at the onset of illness and even in the later stage of the disease, as noted in morbidly obese subjects who achieve weight loss with gastric bypass surgery alone or with hypo-caloric diet, exercise, and addition of oral agents as noted in this study.[5,6] Several physiologic mechanisms may explain this improvement in β-cell function. The role of recovery from inhibition by exogenous insulin, enhancement of insulin sensitivity in the peripheral tissue as well as at the level of β-cell itself, and remission from glucose toxicity in improvement in β-cell function are well established.[1–3,11,26,27,32] Decrease in clearance of C-peptide may be another possible mechanism but is unlikely since both renal and hepatic function remained intact at the time of the repeated OGTT. Alternatively, improvement in the recently recognized decline in the incretin effect in T2DM may have contributed to the reversal of β-cell failure as well.[40] Finally, it is plausible that β-cell failure in T2DM may be an expression of microvascular involvement of the β-cells themselves, with increasing fibrosis resulting in reduction in the number of β-cells as well as deranged function of the remaining cells; in the UKPDS, β-cell failure, as reflected by rising HbA_1c_ (above 7.0%) while on an oral agent, occurred at around the same time as the onset of microvascular complications.[5,16,39] The concept of microvascular disease involving the β-cells themselves received further support recently by the demonstration of progressively rising prevalence of β-cell failure in association with increasing number of microvascular complications and preserved β-cell function in patients without microvascular complications.[41,42] Microvascular disease is attributed to deposition of glycated proteins in organs and tissues. Deposition of amyloid, a glycoprotein, is well known to occur in T2DM.[43] Finally, fibrosis of the pancreatic islets in subjects with T2DM of long duration, which was documented in a recent study, may add credence to this hypothesis.[44] Therefore we believe that β-cell failure could be delayed or prevented by attaining and maintaining glycemic control, which is known to provide beneficial effects with regard to the other well-known microvascular complications in both type 1 and type 2 DM.[5,6,45]
